# Portfolio diet and LDL-C in a young, multiethnic cohort: cross-sectional analyses with cumulative exposure modeling

**DOI:** 10.1186/s12889-025-22479-9

**Published:** 2025-05-13

**Authors:** Victoria Chen, Laura Chiavaroli, Andrea J. Glenn, Meaghan E. Kavanagh, Tara Zeitoun, Sara Mahdavi, Cyril W. C. Kendall, David J. A. Jenkins, Ahmed El-Sohemy, John L. Sievenpiper

**Affiliations:** 1 Department of Nutritional Sciences, Temerty Faculty of Medicine, University of Toronto, Toronto, ON Canada; 2 Toronto 3D Knowledge Synthesis and Clinical Trials Unit, Clinical Nutrition and Risk Factor Modification Centre, St. Michael’s Hospital, Toronto, ON Canada; 3 Department of Nutrition, Harvard T.H. Chan School of Public Health, Boston, MA USA; 4 Department of Nutrition and Food Studies, New York University, New York, NY USA; 5 Department of Medicine, Temerty Faculty of Medicine, University of Toronto, Toronto, ON Canada; 6 Division of Endocrinology and Metabolism, St. Michael’s Hospital, Toronto, ON Canada; 7 Li Ka Shing Knowledge Institute, St. Michael’s Hospital, Toronto, ON Canada; 8 College of Pharmacy and Nutrition, University of Saskatchewan, Saskatoon, SK Canada

**Keywords:** Portfolio Diet, LDL-C, Cardiovascular risk factors, Cumulative exposure, Primordial prevention, Young adults

## Abstract

**Background:**

The Portfolio Diet is a plant-based dietary pattern of cholesterol-lowering foods that has demonstrated clinically meaningful reductions in low-density lipoprotein cholesterol (LDL-C) and other cardiovascular risk factors. However, the Portfolio Diet has not been assessed in an ethnoculturally diverse population of young adults.

**Objective:**

To examine the association of the Portfolio Diet Score (PDS) with LDL-C and other established cardiovascular risk factors in a young adult population.

**Methods:**

This cross-sectional analysis included 1,507 men and women (mean age, 23 ± 3 years) of diverse ethnocultural backgrounds from the Toronto Nutrigenomics and Health Study. Diet was assessed by a validated Toronto-modified Harvard 196-item food frequency questionnaire with adherence to the Portfolio Diet measured using the Portfolio Diet Score. Data were analyzed using multiple linear regressions with adjustment for potential confounders. Modeling analyses related LDL-C levels according to absolute adherence to the Portfolio Diet with cumulative LDL-C and onset of rising cardiovascular risk by age.

**Results:**

Participants were Caucasian (49%), East Asian (34%), South Asian (11%), or other (7%) with a mean LDL-C of 2.3 ± 0.7mmol/L. A 1-point higher PDS and higher PDS tertiles were associated with lower LDL-C (ß [95% CI] per 1-point: -0.009mmol/L [-0.016, -0.002], *P* = 0.013; P_trend_ across tertiles =0.040), non-HDL-C (-0.010mmol/L [-0.018, -0.002], *P* = 0.014; P_trend_=0.028), total cholesterol (-0.011mmol/L [-0.019, -0.003], *P* = 0.011; P_trend_=0.038), systolic blood pressure (-0.150mmHg [-0.250, -0.050], *P* = 0.003; P_trend_<0.001) and diastolic blood pressure (-0.133mmHg [-0.219, -0.046], *P* = 0.003; P_trend_<0.001). Higher PDS tertiles were associated with lower triglycerides (P_trend_=0.039). A 1-point higher PDS was also associated with lower BMI (-0.038 kg/m^2^ [-0.071, -0.004], *P* = 0.026), waist circumference (-0.092cm [-0.171, -0.013], *P* = 0.022), body weight (-0.124 kg [-0.229, -0.019], *P* = 0.021) and FMI (-0.019 kg/m^2^ [-0.037, -0.001], *P* =0.039). There was no association with HDL-C, CRP, or fasting glucose. Modeling analyses suggest that compared to low adherence, 50% and 100% adherence to the Portfolio Diet may delay the onset of rising cardiovascular risk by an estimated 6 and 13 years, respectively.

**Conclusions:**

Among young adults, the PDS was inversely associated with LDL-C and several other established cardiovascular risk factors. Early adherence to the Portfolio Diet may limit lifetime exposure to LDL-C and could delay the age at which cardiovascular events begin.

**Supplementary Information:**

The online version contains supplementary material available at 10.1186/s12889-025-22479-9.

## Introduction

Cardiovascular disease (CVD) continues to be a leading cause of death in Canada, the United States and globally [1, 2]. Recently, the high prevalence of cardiovascular risk factors, including diabetes, obesity, hypertension, and hyperlipidemia, in young adults has become an area of particular concern [3, 4]. Mendelian randomization studies and randomized controlled trials have established the causal role of low-density lipoprotein cholesterol (LDL-C) in CVD incidence [5, 6]. Additionally, longer lifetime exposure to hyperlipidemia has been shown to increase CVD risk in a dose-dependent manner [7–9]. Therefore, early management of hyperlipidemia beginning in young adulthood (an age prior ≥ 30 years of age based on the Framingham Risk Score [10]) represents a key opportunity for CVD prevention [11]. Adoption of healthy lifestyle behaviours may act as an important early management strategy prior to the initiation of pharmacotherapy [11–13].

Dietary modifications remain a cornerstone for the management of hyperlipidemia [12, 13]. A dietary approach specifically developed for cholesterol-lowering is the Portfolio Diet, a therapeutic, plant-based dietary pattern which has demonstrated drug-like reductions in LDL-C [14, 15]. The Portfolio Diet consists of 5 pillars of cholesterol-lowering foods or food components (sources of plant protein, viscous fibre, nuts, phytosterols and monounsaturated fatty acids (MUFAs)) on top a low saturated fat and cholesterol, National Cholesterol Education Program (NCEP) Step II diet [16, 17]. Each pillar of the Portfolio Diet carries an approved health claim for cholesterol-lowering or coronary heart disease risk reduction from Health Canada, the US Food & Drug Administration and/or the European Food Safety Authority [18–25]. Beyond LDL-C, a systematic review and meta-analysis of controlled trials has demonstrated that the Portfolio Diet leads to clinically-meaningful benefits in other established cardiovascular risk factors including total cholesterol, triglycerides, non-high-density lipoprotein (non-HDL-C), systolic blood pressure (SBP), diastolic blood pressure (DBP) and C-reactive protein (CRP) in middle-aged adults (mean age, 57 years) [26]. A prospective cohort study has additionally shown that higher adherence to the Portfolio Diet was associated with improved glycemic control and adiposity markers in older adults (mean age, 65 years) with metabolic syndrome [27]. Notably, findings from a prospective cohort [27] and from RCTs [14, 15] have been in predominantly Caucasian populations (98% and ~80%, respectively). Therefore, there is limited evidence of the role of the Portfolio Diet and cardiovascular risk factors in different ethnic groups and no evidence in young adults. This study aims to assess the association of the Portfolio Diet Score with LDL-C and other established cardiovascular risk factors in an ethnoculturally diverse population of young adults.

## Methods

### Study population

This analysis was conducted within the framework of the Toronto Nutrigenomics and Health (TNH) Study, which has been described elsewhere [28]. Briefly, the TNH Study is a cross-sectional study in young adults in Canada investigating the associations between dietary intake and genetic variation on biomarkers of chronic disease. Participants aged 20–29 years (mean age, 23 ± 3 years) from diverse ethnocultural backgrounds were recruited from the University of Toronto campus between 2004 and 2010. The study protocol was approved by the Research Ethics Board at the University of Toronto.

We excluded participants with implausible energy intakes (> 3500 kcal/day for females, > 4500 kcal/day for males or < 800 kcal/day for females or males) (*n* = 119), with incomplete FFQ data (*n* = 6) or with missing covariates (*n* = 1). Additional exclusions were made for missing outcome data (*n* = 17 for LDL-C, non-HDL-C and HDL-C; *n* = 16 for total cholesterol and triglycerides; *n* = 13 for CRP; *n* = 11 for fasting glucose). The final analysis included between 1,490 and 1,507 participants for individual outcomes.

### Assessment of adherence to the portfolio diet

Dietary intake was collected using a validated Toronto-modified Harvard 196-item semi-quantitative food frequency questionnaire (FFQ) measuring the quantity and frequency of intake over 1-month [29]. Participants were given instructions on how to complete the FFQ using visual aids of portion sizes. Adherence to the Portfolio Diet was assessed using a population-based Portfolio Diet Score (PDS), which was previously developed and validated using the same modified FFQ against 7-day diet records and LDL-C [30]. Food items on the FFQ were categorized into the five components of the Portfolio Diet (sources of plant protein from soy or dietary pulses, viscous fibre such as oats, barley and temperate climate fruit, nuts including nut butters, phytosterols estimated from all plant foods and MUFAs from avocados and olive oil) and saturated fat and cholesterol from animal sources. These six food components were assessed as servings/day, except for phytosterol intake which was assessed as mg/day derived from all plant-based food FFQ items to estimate total intake. Points were assigned for each component based on adherence according to population-based quintiles of intake. Positive points were assigned to foods/nutrients recommended in the diet (sources of plant protein, viscous fibre, nuts, phytosterols and MUFAs), where participants in the highest quintile received five points and those in the lowest quintile received one point. Reverse points were given for foods not recommended in the diet (high saturated fat and cholesterol sources), where participants in the highest quintile received one point and those in the lowest quintile received five points. The six individual component scores were summed to give a PDS between 6 and 30, where a higher score indicates higher adherence to recommendations. Supplementary Table 1 presents the food items in the FFQ, the scoring criteria to determine the PDS, and the average intakes of each component by quintile.

### Assessment of outcomes

The primary outcome was LDL-C, a primary lipid target for CVD prevention [12]. Secondary outcomes included non-HDL-C, total cholesterol, HDL-C, triglycerides, CRP, fasting glucose, SBP, DBP, BMI, waist circumference, body weight and fat mass index (FMI). Fasting blood samples were collected after a 12-hour overnight fast and measured for lipid, glycemic control, and inflammatory markers [31]. LDL-C was calculated using the National Institutes of Health (NIH) equation [32], which has demonstrated high accuracy compared to direct measured and ß-quantification LDL-C against other formulas [32, 33] and is recommended by Canadian guidelines [34]. Sensitivity analyses were conducted using the Martin [35] and Vujovic [36] equations, which have also demonstrated high accuracy [33]. Negative computed LDL-C values were replaced with the minimum positive LDL-C value computed in the sample (*n* = 1). Non-HDL-C was calculated by subtracting HDL-C from total cholesterol. Anthropometric variables including height, weight, waist circumference and blood pressure were measured using standard procedures [28]. BMI was calculated by dividing the weight (kg) by the height squared (m^2^). FMI was calculated by multiplying body fat percentage (%) by body weight (kg) and dividing by the height squared (m^2^). Body fat percentage, used to compute FMI, was calculated using the Deurenberg formula [37].

### Assessment of covariates

Information on participant’s demographics (age, sex, ethnicity, and level of education), lifestyle (physical activity and smoking), medical history (high blood pressure and high cholesterol) and family history (family history of CVD and diabetes) were collected using a general health and lifestyle questionnaire. Family history was defined as having a parent or sibling with CVD or diabetes. Total energy and alcohol intake were assessed by the FFQ.

### Statistical analyses

Descriptive analyses across tertiles of the PDS were conducted using the analysis of variance (ANOVA) for continuous variables and chi-square test for categorical variables. Characteristics were presented as means and standard deviations for continuous variables and frequencies with percentages for categorical variables. Data were analysed using multiple linear regressions to estimate ß-coefficients and 95% confidence intervals for the associations of the PDS with LDL-C and other established cardiovascular risk factors (non-HDL-C, total cholesterol, HDL-C, triglycerides, CRP, fasting glucose, SBP, DBP, BMI, waist circumference, body weight and FMI). We examined associations of outcomes continuously per 1- and per 8-PDS points. We also examined associations of outcomes by tertiles of the PDS (lower, medium, and higher adherence), where tests for linear trend were conducted by assigning the median PDS value to each tertile. Marginal means with 95% confidence intervals at each tertile were presented. As the PDS is a single composite score of its components, multicollinearity and interactions between individual dietary components were not considered in models assessing the association between the PDS and cardiovascular risk factors. Additional multiple linear regression analyses were conducted to assess PDS components individually by servings of intake (a serving of phytosterols was estimated to be 25 mg from the mean phytosterol content of plant foods included in the FFQ). Assumptions of normality were verified by visual inspection of quantile-quantile plots for all outcomes and continuous covariates. All outcomes and continuous covariates were normally distributed, except for triglycerides, CRP, BMI, waist circumference, body weight, FMI and alcohol intake, which were log-transformed or square root-transformed to an approximately normal distribution before analysis. For models using log-transformed outcome variables, non-transformed marginal means or exponentiated ß coefficients multiplied by the non-transformed mean, to estimate relative proportional differences, were reported to facilitate interpretation.

Models were adjusted for sex (male, female), age (continuous), education (high school, some college/university, college/university degree, graduate degree), ethnicity (Caucasian, East Asian, South Asian and other [individuals who reported belonging to ≥ 2 ethnocultural groups not included in the same category, Aboriginal Canadians, or Afro-Caribbeans]), BMI (continuous), family history of CVD (yes/no), family history of diabetes (yes/no), hypertension status (yes/no), hypercholesterolemia status (yes/no), energy intake (continuous), smoking (current smoker [≥ 1/day], non-smoker), physical activity (continuous) and alcohol intake (continuous). A priori subgroup analyses were conducted by examining for potential interactions with sex, ethnicity, and categorical BMI (< 25 kg/m^2^, ≥ 25 kg/m^2^). All analyses were performed with STATA version 17.0 (StatCorp, College Station, TX, USA) or R version 4.2.1 (R Foundation for Statistical Computing) and RStudio. Statistical significance was set at a P-value < 0.05.

Although cardiovascular risk in young adulthood tends to be low, lifetime risk depends on both absolute magnitude and cumulative exposure to LDL-C [5], therefore the early maintenance of LDL-C levels using dietary interventions may impact cardiovascular health in later life [38]. To explore the long term impact of adherence to the Portfolio Diet in young adulthood, we modeled cumulative LDL-C exposure. We related absolute adherence to calculated cumulative LDL-C exposure, plaque burden and onset of cardiovascular risk by age, using previously published formulas and thresholds [38]. Specifically, food items on the FFQ in each Portfolio Diet pillar were converted to g/day of plant protein, viscous fibre, nuts and seeds, phytosterols and MUFAs to assess absolute adherence to Portfolio Diet recommendations in each tertile of the PDS [14–17, 39, 40]. Absolute adherence was presented as percentages and as points according to the clinical-Portfolio Diet Score (c-PDS), a food-based 25-point score based on a 2000kcal/day diet [41]. Absolute adherence was related to unadjusted marginal mean LDL-C levels in each PDS tertile determined in our main analysis. LDL-C at other levels of adherence was estimated linearly, based on previous evidence [14, 15]. Cumulative LDL-C was calculated by multiplying age by LDL-C at each level of adherence [38]. Using this formula, we estimated age at which cumulative LDL-C reached 125 mmol-years, the threshold which marks sufficiently large plaque burden and onset of exponentially increasing myocardial infarction risk [38]. The key assumptions underlying this approach are that estimated LDL-C levels, based on observed associations, are constant over time, and the impact of other cardiovascular disease risk factors and potential covariates are maintained over the modeled period.

## Results

### Participant characteristics

Table 1 shows the participant characteristics across tertiles of the PDS. Those with higher PDS were more likely to be older, female, more physically active, have higher total energy intake, drink more alcohol, and have a college/university degree. PDS also differed between ethno-cultural groups, with relatively more Caucasians in the highest tertile of adherence. Supplementary Table 1 shows mean intakes of each PDS component.

**Table 1:** Participant characteristics of the 1,507 TNH study participants according to tertiles of the portfolio diet score

	Portfolio Diet Score (PDS)		
	T1 (low, 6–15)	T2 (medium, 16–20)	T3 (high, 21–30)
*n*	528	537	442
Median PDS	13	18	23
Age, y	22.4 (2.4)	22.6 (2.5)	23.2 (2.6)^a, b^
Sex, female, *n* (%)	339 (64)	369 (69)	324 (73)^a^
BMI, kg/m^2^	23.0 (3.9)	23.1 (3.6)	22.7 (3.2)
Ethnicity, *n* (%)^c^			
Caucasian	221 (42)	253 (47)	257 (58)
East Asian	197 (37)	187 (35)	123 (28)
South Asian	67 (13)	55 (10)	38 (9)
Other	43 (8)	42 (8)	24 (5)
Physical activity, PAL	1.71 (0.38)	1.77 (0.40)^a^	1.80 (0.37)^a^
Total energy intake, kcal/day	1740 (594)	1975 (632)^a^	2242 (634)^a, b^
Alcohol intake, g/d	5.4 (10.7)	5.6 (9.0)	6.2 (7.8)^a^
Family history of CVD, *n* (%)	4 (1)	9 (2)	11 (3)
Family history of diabetes, *n* (%)	83 (16)	61 (11)	47 (11)
Education, *n* (%)^c^			
High school	12 (2)	6 (1)	9 (2)
Some college/university	326 (62)	331 (62)	221 (50)
College/university	154 (29)	153 (29)	158 (36)
Graduate	36 (7)	47 (9)	54 (12)
High blood pressure, *n* (%)	7 (1)	5 (1)	4 (1)
High cholesterol, *n* (%)	19 (4)	20 (4)	10 (2)

Data are shown as mean (SD) or *n* (%)

T1 (low), T2 (mid), and T3 (highest) tertiles of adherence to the Portfolio dietary pattern measured using the Portfolio Diet Score (PDS)

BMI, body mass index; CVD, cardiovascular disease; PAL, physical activity level; SD, standard deviation; T, tertile; TNH, Toronto Nutrigenomics and Health

^a^ significantly different from T1 (< 0.05)

^b^ significantly different from T2 (< 0.05)

^c^ significantly different distributions across tertiles (< 0.05)

### Portfolio diet and established cardiovascular risk factors

Table 2 shows the associations between the PDS and cardiovascular risk factors. After multivariable adjustments, a 1-point higher PDS was associated with lower LDL-C (ß [95% CI]: -0.009mmol/L [-0.016, -0.002], *P* = 0.013). An inverse association between higher PDS tertiles and LDL-C was also observed (P_trend_=0.040). The use of the Martin or Vujovic equations to calculate LDL-C did not alter the significance or magnitude of the results (Supplementary Table 2). A 1-point higher PDS and higher PDS tertiles were additionally associated with several secondary outcomes including non-HDL-C (-0.010mmol/L [-0.018, -0.002], *P* = 0.014; P_trend_=0.028), total cholesterol (-0.011mmol/L [-0.019, -0.003], *P* = 0.011; P_trend_=0.038), SBP (-0.150mmHg [-0.250, -0.050], *P* = 0.003; P_trend_<0.001) and DBP (-0.133mmHg [-0.219, -0.046], *P* = 0.003; P_trend_<0.001). While there was a significant association between higher PDS tertiles and lower triglycerides (P_trend_=0.039), this association was not significant in the continuous analysis per-PDS point, although it tended toward an inverse relationship. Continuous analyses demonstrated a significant association between a 1-point higher PDS and lower BMI (-0.038 kg/m^2^ [-0.071, -0.004], *P* = 0.026), waist circumference (-0.092cm [-0.171, -0.013], *P* = 0.022), body weight (-0.124 kg [-0.229, -0.019], *P* = 0.021) and FMI (-0.019 kg/m^2^ [-0.037, -0.001], *P* = 0.039). The P-trends across PDS tertiles were not significant for BMI, waist circumference, body weight and FMI; however, the these markers of adiposity tended to be lower with higher tertiles. We did not observe associations between PDS and HDL-C, CRP, or fasting glucose.

**Table 2:** Cardiovascular risk factors according to tertiles and points of the portfolio diet score

	*n*	T1 (low)	T2 (medium)	T3 (high)	*P*-trend^a^	Per 1-PDS point	Per 8-PDS points	*P*-value^b^
Primary outcome								
**LDL-C (mmol/L)**								
Unadjusted model	1,490	2.34 (2.28, 2.39)	2.29 (2.25, 2.32)	2.24 (2.18, 2.29)	**0.023**	-0.010 (-0.017, -0.003)	-0.078 (-0.133, -0.023)	**0.006**
Multivariable model	1,490	2.60 (2.36, 2.84)	2.55 (2.32, 2.79)	2.51 (2.27, 2.75)	**0.040**	-0.009 (-0.016, -0.002)	-0.073 (-0.130, -0.015)	**0.013**
Secondary outcomes								
**Lipids**								
**Non-HDL-C (mmol/L)**								
Unadjusted model	1,490	2.76 (2.70, 2.82)	2.70 (2.67, 2.74)	2.65 (2.59, 2.71)	**0.016**	-0.011 (-0.019, -0.003)	-0.087 (-0.149, -0.026)	**0.005**
Multivariable model	1,490	3.09 (2.83, 3.35)	3.04 (2.78, 3.29)	2.98 (2.72, 3.25)	**0.028**	-0.010 (-0.018, -0.002)	-0.079 (-0.143, -0.016)	**0.014**
**Total cholesterol (mmol/L)**								
Unadjusted model	1,491	4.28 (4.22, 4.34)	4.25 (4.21, 4.28)	4.21 (4.14, 4.27)	0.128	-0.008 (-0.016, 0.000)	-0.066 (-0.130, -0.001)	**0.045**
Multivariable model	1,491	4.51 (4.24, 4.79)	4.46 (4.19, 4.73)	4.40 (4.13, 4.68)	**0.038**	-0.011 (-0.019, -0.003)	-0.087 (-0.154, -0.020)	**0.011**
**HDL-C (mmol/L)**								
Unadjusted model	1,490	1.52 (1.49, 1.55)	1.54 (1.52, 1.56)	1.56 (1.53, 1.59)	0.101	0.003 (-0.001, 0.007)	0.022 (-0.010, 0.055)	0.177
Multivariable model	1,490	1.42 (1.30, 1.55)	1.42 (1.30, 1.55)	1.42 (1.30, 1.55)	0.943	-0.001 (-0.005, 0.003)	-0.008 (-0.038, 0.022)	0.614
**Triglycerides (mmol/L)** ^**c**^								
Unadjusted model	1,491	1.00 (0.96, 1.05)	0.97 (0.94, 1.00)	0.94 (0.89, 0.99)	**0.020**	-0.005 (-0.010, -0.001)	-0.042 (-0.076, -0.008)	**0.017**
Multivariable model	1,491	1.16 (0.95, 1.37)	1.13 (0.92, 1.34)	1.10 (0.89, 1.31)	**0.039**	-0.005 (-0.009, 0.000)	-0.037 (-0.073, 0.000)	0.051
**Inflammation**								
**CRP (mg/L)** ^**c**^								
Unadjusted model	1,494	1.40 (1.20, 1.61)	1.26 (1.31, 1.40)	1.12 (0.91, 1.34)	0.395	-0.008 (-0.023, 0.007)	-0.064 (-0.174, 0.057)	0.287
Multivariable model	1,494	1.40 (1.19, 1.60)	1.26 (1.14, 1.39)	1.13 (0.91, 1.35)	0.297	-0.009 (-0.024, 0.006)	-0.070 (-0.177, 0.048)	0.236
**Glycemic control**								
**Fasting glucose (mmol/L)**								
Unadjusted model	1,496	4.80 (4.78, 4.83)	4.79 (4.77, 4.81)	4.77 (4.74, 4.80)	0.154	-0.004 (-0.007, 0.000)	-0.028 (-0.058, 0.003)	0.075
Multivariable model	1,496	4.81 (4.68, 4.94)	4.81 (4.68, 4.94)	4.81 (4.68, 4.94)	0.830	-0.001 (-0.005, 0.003)	-0.006 (-0.038, 0.025)	0.688
**Blood pressure**								
**SBP (mmHg)**								
Unadjusted model	1,507	115 (114, 116)	114 (113, 115)	113 (112, 114)	**< 0.001**	-0.170 (-0.291, -0.049)	-1.361 (-2.332, -0.390)	**0.006**
Multivariable model	1,507	124 (121, 127)	123 (119, 126)	121 (118, 125)	**< 0.001**	-0.150 (-0.250, -0.050)	-1.202 (-2.002, -0.402)	**0.003**
**DBP (mmHg)**								
Unadjusted model	1,507	70.2 (69.6, 70.8)	69.3 (68.9, 69.7)	68.4 (67.8, 69.1)	**< 0.001**	-0.128 (-0.212, -0.043)	-1.021 (-1.695, -0.347)	**0.003**
Multivariable model	1,507	73.8 (70.9, 76.6)	72.8 (70.0, 75.6)	71.8 (69.0, 74.7)	**< 0.001**	-0.133 (-0.219, -0.046)	-1.061 (-1.755, -0.367)	**0.003**
**Markers of adiposity**								
**BMI (kg/m**^**2**^) ^**c**^								
Unadjusted model	1,507	23.1 (22.8, 23.3)	22.9 (22.7, 23.1)	22.8 (22.5, 23.1)	0.375	-0.024 (-0.059, 0.011)	-0.189 (-0.465, 0.090)	0.184
Multivariable model	1,507	25.1 (23.9, 26.3)	24.9 (23.8, 26.1)	24.7 (23.6, 25.9)	0.131	-0.038 (-0.071, -0.004)	-0.299 (-0.560, -0.036)	**0.026**
**Waist circumference (cm)** ^**c**^								
Unadjusted model	1,507	74.8 (74.1, 75.6)	74.3 (73.8, 74.8)	73.8 (73.0, 74.6)	0.134	-0.086 (-0.177, 0.005)	-0.685 (-1.406, 0.044)	0.065
Multivariable model	1,507	81.9 (79.1, 84.7)	81.5 (78.7, 84.2)	81.0 (78.2, 83.8)	0.107	-0.092 (-0.171, -0.013)	-0.734 (-1.355, -0.107)	**0.022**
**Body weight (kg)** ^**c**^								
Unadjusted model	1,507	65.1 (64.0, 66.1)	64.4 (63.8, 65.1)	63.8 (62.7, 64.9)	0.292	-0.079 (-0.209, 0.052)	-0.627 (-1.656, 0.419)	0.239
Multivariable model	1,507	73.2 (69.4, 76.9)	72.4 (68.7, 76.1)	71.7 (67.9, 75.4)	0.075	-0.124 (-0.229, -0.019)	-0.987 (-1.810, -0.154)	**0.021**
**FMI (kg/m**^**2**^) ^**c**^								
Unadjusted model	1,507	5.58 (5.40, 5.75)	5.61 (5.50, 5.72)	5.65 (5.46, 5.83)	0.132	0.013 (-0.009, 0.034)	0.102 (-0.069, 0.279)	0.243
Multivariable model	1,507	6.48 (5.79, 7.17)	6.35 (5.67, 7.03)	6.22 (5.52, 6.92)	0.141	-0.019 (-0.037, -0.001)	-0.152 (-0.291, -0.008)	**0.039**

Associations between the PDS (ranging from 6-30 points) and cardiovascular risk factors by tertiles of the PDS (lower, medium and higher adherence) and per 1- and per 8-PDS points were assessed using multiple linear regressions. Data are marginal means (95% CIs) for each tertile and ß-coefficients (95% CIs) for continuous analyses by points. Prior to analysis, non-normal outcomes and covariates (triglycerides, CRP, BMI, waist circumference, body weight, FMI and alcohol intake) were log-transformed or square-root transformed to an approximately normal distribution. Multivariable models were adjusted for sex (male, female), age (continuous), education (high school, some college/university, college/university degree, graduate degree), ethnicity (Caucasian, East Asian, South Asian and other), BMI (continuous; not included in models for markers of adiposity), family history of CVD (yes/no), family history of diabetes (yes/no), hypertension status (yes/no), hypercholesterolemia status (yes/no), energy intake (continuous), smoking (current smoker [≥ 1/day], non-smoker), physical activity (continuous) and alcohol intake (continuous)

T1 (low), T2 (mid), and T3 (highest) tertiles of adherence to the Portfolio dietary pattern measured using the Portfolio Diet Score (PDS)

BMI, body mass index; CRP, c-reactive protein; DBP, diastolic blood pressure; FMI, fat mass index; HDL-C, high-density lipoprotein cholesterol; LDL-C, low-density lipoprotein cholesterol; PDS, Portfolio Diet Score; SBP, systolic blood pressure; T, tertile

To convert LDL-C, non-HDL-C, total cholesterol or HDL-C from mmol/L to mg/dL multiply by 38.67

To convert triglycerides from mmol/L to mg/dL multiply by 88.57

To convert CRP from mg/L to mg/dL divide by 10

To convert fasting glucose from mmol/L to mg/dL multiply by 18.018

^a^ P for trend obtained from assigning the median value to each tertile

^b^ P value obtained from continuous point increases in ß-coefficients

^c^ Non-transformed marginal means or exponentiated ß coefficients multiplied by the non-transformed mean, were reported to facilitate interpretation of log-transformed variables. P-values provided are from the log-transformed analysis

### Subgroup analyses

Supplementary Tables 3–15 show the subgroup analyses by sex, ethnicity, and BMI for all outcomes. After multivariable adjustments, there were interactions by sex and ethnicity for total cholesterol and fasting glucose, respectively. The association between PDS and lower total cholesterol was stronger and significant in males only (Males: -0.023mmol/L [-0.038, -0.008]; Females: -0.006 [-0.016, 0.004], P_interaction_=0.041) (Supplementary Table 5). The association of PDS and lower fasting glucose was stronger in East Asians and South Asians, although significant in East Asians only (Caucasian: 0.003mmol/L [-0.002, 0.008]; East Asian: -0.008 [-0.015, -0.0002]; South Asian: -0.013 [-0.027, 0.0003]; Other: 0.015 [-0.002, 0.032], P_interaction_=0.006) (Supplementary Table 9).

### Individual portfolio diet score component analyses

Supplementary Table 16 shows the associations between the individual components of the PDS and cardiovascular risk factors. After multivariable adjustments, higher intake of sources of plant protein, nuts and phytosterol was associated with lower LDL-C (plant protein: -0.051mmol/L [-0.090, -0.011], *P* = 0.012; nuts: -0.049 [-0.096, -0.003], *P* = 0.038; phytosterols: -0.013 [-0.021, -0.005], *P* = 0.002), non-HDL-C (plant protein: -0.054mmol/L [-0.097, -0.010], *P* = 0.016; nuts: -0.062 [-0.113, -0.010], *P* = 0.019; phytosterols: -0.015 [-0.024, -0.006], *P* = 0.001) and total cholesterol (plant protein: -0.057mmol/L [-0.103, -0.011], *P* = 0.016; nuts: -0.057 [-0.111, -0.002], *P* = 0.041; phytosterols: -0.017 [-0.026, -0.007], *P* = 0.001). Higher intake of sources of saturated fat and cholesterol was associated with higher LDL-C (0.028mmol/L [0.004, 0.051], *P* = 0.020), total cholesterol (0.036mmol/L [0.009, 0.063], *P* = 0.010) and HDL-C (0.013mmol/L [0.001, 0.025], *P* = 0.039). Higher intake of sources of nuts, MUFAs and phytosterols was associated with lower triglycerides (nuts: -0.040mmol/L [-0.069, -0.010], *P* = 0.009; MUFAs: -0.033mmol/L [-0.063, -0.002], *P* = 0.036; phytosterols: -0.006mmol/L [-0.012, -0.001], *P* = 0.020). Higher intake of sources of MUFAs was also associated with lower CRP (-0.096 mg/L [-0.183, -0.001], *P* = 0.048).

Higher intake of sources of saturated fat and cholesterol was associated with higher systolic (0.451mmHg [0.128, 0.774], *P* = 0.006) and diastolic (0.345mmHg [0.064, 0.625], *P* = 0.016) blood pressure. Higher intake of sources of plant protein was associated with lower systolic blood pressure (-0.854mmHg [-1.403, -0.305], *P* = 0.002) and higher intake of sources of viscous fibre was associated with lower diastolic blood pressure (-0.375mmHg [-0.723, -0.027], *P* = 0.035).

For markers of adiposity, higher intake of sources of nuts and phytosterols was associated with lower BMI (nuts: -0.359 kg/m^2^ [-0.568, -0.147], *P* = 0.001; phytosterols: -0.048 [-0.085, -0.010], *P* = 0.013), waist circumference (nuts: -0.827cm [-1.327, -0.324], *P* = 0.001; phytosterols: -0.108 [-0.198, -0.019], *P* = 0.017), body weight (nuts: -0.754 kg [-1.421, -0.080], *P* = 0.029; phytosterols: -0.171 [-0.290, -0.052], *P* = 0.005) and FMI (nuts: -0.199 kg/m^2^ [-0.311, -0.085], *P* = 0.001; phytosterols: -0.025 [-0.046, -0.004], *P* = 0.018).

### Portfolio diet and cardiovascular risk onset

**Fig. 1:**
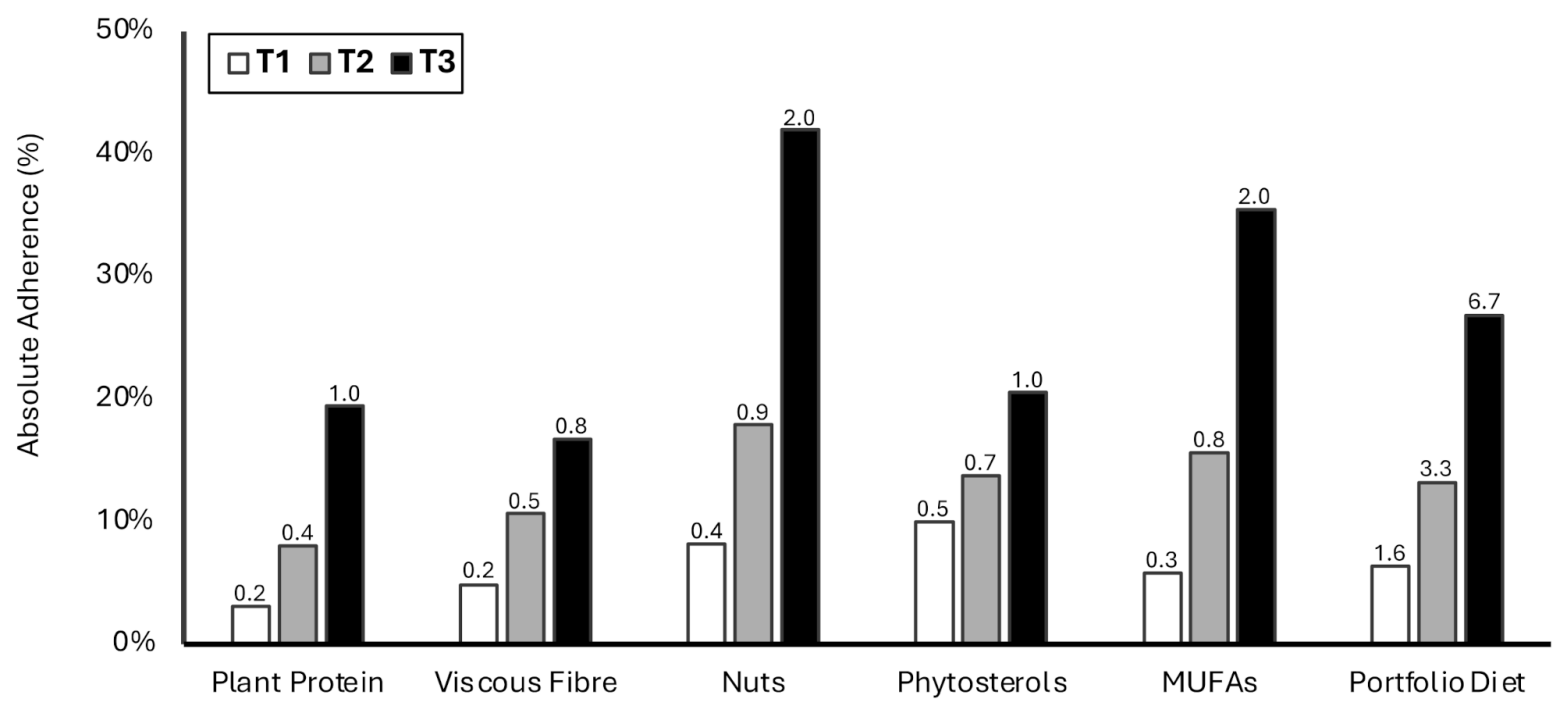
Absolute adherence to Portfolio Diet recommendations according to tertiles of the Portfolio Diet Score. Food items on the FFQ in each Portfolio Diet pillar were converted to g/day of plant protein, viscous fibre, nuts, phytosterols and MUFAs and compared to Portfolio Diet recommendations to assess absolute adherence in each tertile of the Portfolio Diet Score (PDS). Adherence was based on established Portfolio Diet recommendations (50g/day plant protein, 20g/day viscous fibre, 45g/day nuts, 2g/day phytosterols and 45g/day MUFAs). Absolute adherence to recommendations is presented as percentage adherence on the y-axis. Absolute adherence according to the clinical-Portfolio Diet Score (c-PDS) score is shown above each bar. The c-PDS score gives 0 to 5 points for each component, yielding a total c-PDS between 0 and 25. The white, grey and black bars represent adherence in tertile 1, 2 and 3, respectively. T1 (low), T2 (mid), and T3 (highest) tertiles of adherence to the Portfolio dietary pattern measured using the PDS. c-PDS, clinical-Portfolio Diet Score; MUFAs, monounsaturated fatty acids; T, tertile

Figure 1 shows the absolute adherence to Portfolio Diet recommendations in each PDS tertile. In tertile 1, the absolute adherence to recommendations was 3% for plant protein, 5% for viscous fibre, 8% for nuts, 10% for phytosterols and 6% for MUFAs. In tertile 2, the absolute adherence to recommendations was 8% for plant protein, 11% for viscous fibre, 18% for nuts, 14% for phytosterols and 16% for MUFAs. In tertile 3, the absolute adherence to recommendations was 20% for plant protein, 17% for viscous fibre, 42% for nuts, 21% for phytosterols and 36% for MUFAs. Overall adherence to the Portfolio Diet was 6% (c-PDS, 2), 13% (c-PDS, 3) and 27% (c-PDS, 7) for tertiles 1, 2 and 3, respectively.

**Fig. 2:**
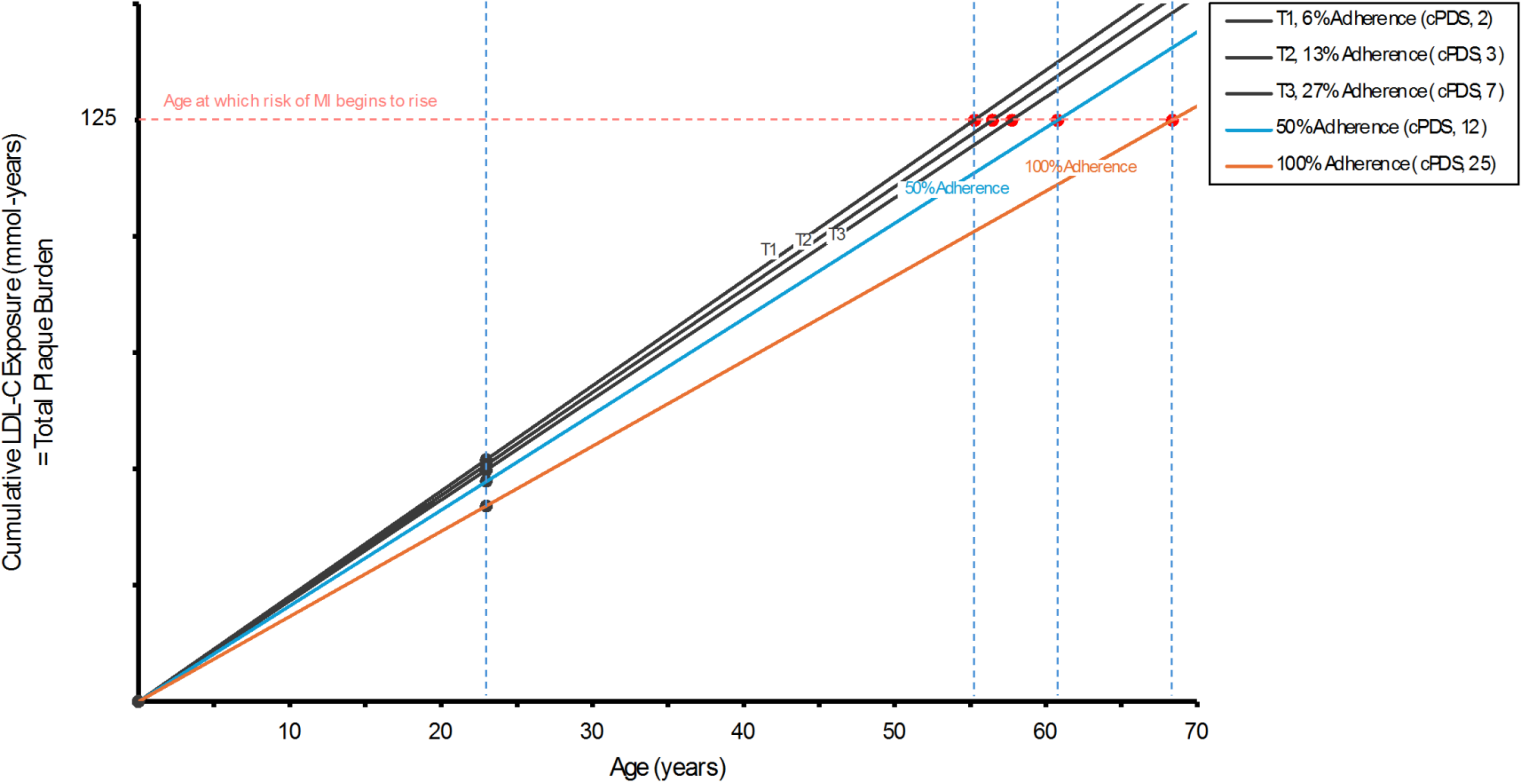
Cumulative LDL-C exposure, plaque burden and onset of cardiovascular risk by age according to absolute adherence to the Portfolio Diet. Absolute adherence was related to unadjusted marginal mean LDL-C levels in each PDS tertile determined in our main analysis. LDL-C at other levels of adherence was estimated linearly. The solid black lines represents tertiles 1, 2 and 3 relating to LDL-C levels of 2.34, 2.29 and 2.24mmol/L throughout life, respectively. The solid blue line represents 50% absolute adherence to Portfolio Diet recommendations relating to an estimated LDL-C level of 2.13mmol/L throughout life. The solid orange line represents 100% absolute adherence to Portfolio Diet recommendation relating to an estimated LDL-C level of 1.89mmol/L throughout life. Cumulative LDL-C exposure was calculated by multiplying LDL-C levels by age. The black dots represent the cumulative LDL-C level at 23 years of age, equal to the mean age of the TNH Study cohort. The red dots represent the average age where cumulative LDL-C reaches 125mmol-years marking the threshold for the onset of exponential myocardial infarction risk increase. Age at which risk of myocardial infarction begins to rise is 55 years for 6% adherence (T1), 61 years for 50% adherence and 68 years for 100% adherence. Modeled using methods from Ference BA et al. J Am Coll Cardiol 2018; 72(10):1141-56 [38]. T1 (low), T2 (mid), and T3 (highest) tertiles of adherence to the Portfolio dietary pattern measured using the Portfolio Diet Score (PDS). c-PDS, clinical-Portfolio Diet Score; LDL-C, low-density lipoprotein cholesterol; MI, myocardial infarction; T, tertile

Figure 2 shows the cumulative association of LDL-C on cardiovascular risk onset by age modeled based on adherence to the Portfolio Diet. Assuming LDL-C levels are held constant, the age at which risk of myocardial infarction is estimated to begin to rise is 55, 56 and 58 years old in PDS tertiles 1, 2 and 3, respectively. If 50% absolute adherence to Portfolio Diet recommendations is achieved, LDL-C is estimated to be 2.13mmol/L and age at cardiovascular risk onset is modeled to be 61 years old. If 100% absolute adherence to Portfolio Diet recommendations is achieved, LDL-C is estimated to be 1.89mmol/L and age at cardiovascular risk onset is modeled to be 68 years old.

## Discussion

In this cross-sectional analysis of young men and women, an 8-point higher PDS was associated with 3% lower LDL-C, the primary outcome. Higher intake of Portfolio Diet components, including sources of plant protein, nuts and phytosterols and lower intake of sources of saturated fat and cholesterol, was also associated with lower LDL-C. Higher PDS was associated with several other established cardiovascular risk factors including, non-HDL-C, total cholesterol, SBP and DBP. There were inverse associations with triglycerides across PDS tertiles and BMI, waist circumference, body weight and FMI per PDS point. There were interactions by sex and ethnicity on the association of the PDS with total cholesterol and fasting glucose, respectively.

### Findings in context of the literature

These findings are largely consistent with previous research assessing the Portfolio Diet with established cardiovascular outcomes. Results of RCTs on the Portfolio Diet and cardiovascular risk factors have demonstrated LDL-C reductions of approximately 30% as well as meaningful reductions in several secondary outcomes, including non-HDL-C, apolipoprotein B, total cholesterol, triglycerides, SBP, DBP and CRP [26]. In our analyses, higher adherence to the Portfolio Diet was associated with lower LDL-C, non-HDL-C, total cholesterol, SBP and DBP in analyses both by tertiles of PDS and by PDS points. Higher tertiles of the PDS were significantly associated with lower triglycerides and a 1-point higher PDS also tended toward lower triglycerides. Evidence from observational studies assessing the PDS with LDL-C has been more inconsistent. Previous longitudinal and cross-sectional analyses of cohorts did not observe significant inverse associations between higher PDS and LDL-C [27, 42]. However, a significant inverse association was seen between higher PDS and change in LDL-C in the PDS validation study, that utilized the same modified FFQ as the current analyses which allows for more Portfolio Diet foods to be captured [30]. Evidence from both RCTs and cross-sectional analyses of cohort studies have shown inverse relationships between the Portfolio Diet and various inflammatory markers [26, 42]. A previous longitudinal cohort analysis also demonstrated a significant favourable association between PDS and markers of glycemic control and adiposity [27]. Our analyses demonstrated a significant association between a 1-point higher PDS with lower BMI, waist circumference, body weight and FMI; however, we did not observe associations for CRP or fasting glucose. These discrepancies may be due to lower adherence to Portfolio Diet recommendations in the current cohort compared to RCTs with a Portfolio Diet intervention [26], or the overall better cardiometabolic profile and younger age of the current population, in comparison to those included in other Portfolio Diet RCTs and cohort studies [26, 27, 42]. It is important to note that, participants included in the previous RCTs (mean age, 57 years) [26] and prospective cohort studies (mean age, 44 years) [27, 30, 42] on the Portfolio Diet were generally middle-aged and ~20–30 years older than the TNH Study cohort.

While our study is the first to investigate the Portfolio Diet in an ostensibly healthy young adult population, the cardiovascular benefits of some individual Portfolio Diet components, including nuts [43, 44], soy protein [45, 46], viscous fibre sources [47] and MUFAs [48, 49] has been demonstrated in randomized trials of similar populations. Our findings are also in agreement with cross-sectional analyses of other dietary patterns that share some similar components (legumes, whole grains, nuts, plant oils, fruits, and vegetables) to the Portfolio Diet, including the Dietary Approaches to Stop Hypertension (DASH) and Mediterranean diets, which have been associated with more favourable lipid, blood pressure and/or adiposity markers in young adults who are predominantly university students [50–53]. A previous longitudinal analysis of the Portfolio and DASH diets found that greater adherence to either diet was associated with favourable changes in triglycerides, HbA1c, fasting glucose, BMI and waist circumference in older adults with metabolic syndrome [27]. Long-term prospective cohort studies have further shown that higher adherence to the DASH and Mediterranean diets in younger adulthood was associated with lower risk of incident CVD, mortality or metabolic syndrome in middle age [54, 55]. Previous prospective cohort studies of the Portfolio Diet have demonstrated an association between PDS and lower risk of CVD, type 2 diabetes, and mortality in middle-aged and older adults [42, 56–58]. The association between higher PDS and lower risk of CVD was stronger among younger (< 60 years) compared to older (≥ 60 years) participants in one study [42]. Further investigation of how these associations may differ with adherence beginning in even younger age groups is warranted.

Although the ethnicity and sex-specific differences in our analyses were minimal, they may be noteworthy as other related subgroup differences have been observed in nutrition studies. We found stronger associations between PDS and lower fasting glucose in East Asians and South Asians. This finding is in agreement with a previous prospective cohort study which showed that an overall healthier diet predicted by the dietary diabetes risk reduction score was associated with a larger absolute risk difference for type 2 diabetes in minority women, inclusive of Asian, Hispanic and Black women, compared to non-Hispanic White women [59]. Our subgroup analyses also showed that the PDS was significantly associated with lower total cholesterol in males only. In line with this finding, the effect of the Mediterranean diet on cardiometabolic outcomes has been shown to be stronger in males compared to females in some studies [60, 61]. However, the opposite has been demonstrated in a longitudinal cohort study where the association of the Portfolio Diet and lipid markers was stronger in females [27]. These variations illustrate the importance of further exploring sex-specific differences related to the Portfolio Diet and to other dietary patterns.

In context with multiple lines of evidence which demonstrate increased risk of CVD with longer lifetime exposure to elevated LDL-C [7–9], we demonstrated that lower LDL-C levels, associated with higher adherence to the Portfolio Diet, may delay the onset of rising myocardial infarction risk. If 100% adherence was achieved in our cohort, estimated LDL-C levels were modeled to be 1.89mmol/L or 73.2mg/dL, which is below the 80mg/dL threshold for ideal cardiovascular health [38]. If this LDL-C level is maintained, myocardial infarction risk was estimated to rise beginning at age 68 compared to at age 55 if LDL-C levels in PDS tertile 1 (6% adherence) were maintained instead. This level of adherence has been demonstrated in metabolic trials on the Portfolio Diet where approximately 100% adherence to recommendations was achieved [14]. In a previous 6-month trial of effectiveness on the Portfolio Diet, where only dietary advice was provided, participants attained a much more modest adherence of 40–50% [15]. If a similar 50% adherence was achieved in this cohort, age at which risk of myocardial infarction begins to rise was estimated to still be delayed by 6 years compared to what is observed for PDS tertile 1. Although these findings suggest that early adherence to the Portfolio Diet may be associated with later life cardiovascular health, these modeled projections are based on cross-sectional associations. This underscores the need for future longitudinal analyses on the Portfolio Diet which begin in young adulthood to further examine these relationships.

### Strengths and limitations

The strengths of our analysis include its ethnoculturally diverse population and the measurement of established risk factors. However, this study is not without limitations. First, the study population consisted of self-selected young adults recruited from a large urban university campus and results may not be representative of the general population. However, the homogeneity of the population may reduce the impact of unmeasured differences and residual confounding of the true associations. Second, the cohort sample size was small, limiting assessment of range of exposure and precision of estimates, particularly in subgroup analyses by ethnicity where only 11% of participants were South Asian (*n* = 160) and 7% were other (*n* = 109). Third, dietary intake was self-reported using an FFQ which, although validated in this population and measured over 1-month [29], may result in measurement errors [62]. Additionally, although the PDS has been previously validated with the same FFQ used to assess dietary intake in the current cohort [30], the score has not been specifically validated for the current population. Fourth, the consumption of Portfolio Diet components was generally low, even in the top quintiles of intake, which may have led to an underestimation of the strength of the observed associations. Due to the availability of data, we were unable to assess the intake of some Portfolio Diet foods, including plant sterol enriched margarine and high fibre cereals, although their intakes were likely minimal. Fifth, the cumulative exposure modeling analysis assumed that estimated LDL-C levels were held constant over time, as only a single measure of LDL-C was available. While the use of multiple LDL-C measurements are needed to improve the reliability of our projections, and future longitudinal analyses are necessary to confirm our findings, previous evidence suggests that a single lipid measurement taken in young adulthood may still be highly predictive of cumulative LDL-C and cardiovascular disease risk later in life [63]. Lastly, although adjustments were made for potential confounders, inherent to the cross-sectional nature of our study temporality cannot be assessed, residual confounding cannot be ruled out, and therefore causality cannot be established. Prospective studies are needed to confirm the observed associations.

### Implications

Our analysis found inverse associations between the PDS and LDL-C, along with other established cardiovascular risk factors, contributing to the current body of evidence on the use of the Portfolio Diet. While these findings may not be clinically significant, based on minimally important differences [26, 64], in older cohorts, the small beneficial associations observed may still be relevant given that they were shown in an already healthy, young, and low-risk population. Furthermore, LDL-C levels were approximately 0.1mmol/L, or 4%, lower among those in the highest compared to lowest tertiles of adherence to the Portfolio Diet, which is comparable to the minimum lipid-lowering seen for every dose doubling of statin drug [65], suggesting that with larger differences in adherence to the diet a meaningful association with LDL-C may be observed even in young adults. As the emphasis on primordial and primary prevention of CVD continues to grow, the early promotion of healthy dietary and lifestyle behaviours, is imperative [66–68]. Healthy dietary interventions in young adulthood may be particularly important, as life transitions most often experienced in this age group, including leaving home and leaving education, have been associated with negative impacts on diet quality [69]. The generally low adherence to the Portfolio Diet seen in our cohort and others [27, 30, 42], highlights that even minimal intake of the Portfolio Diet components may be associated with more favourable risk factors. The benefit of partial adoption has also been demonstrated in a 6-month trial where meaningful reductions in LDL-C were seen with only 40–50% adherence to Portfolio Diet recommendations [15]. In our modeling of the potential impact of adherence to the Portfolio Diet in young adulthood on cardiovascular health in later life, we demonstrated an estimated extension of cardiovascular health span of 6 and 13 years with 50% and 100% Portfolio Diet adherence, respectively. This finding further demonstrates the clinical importance of dietary modification as part of primordial prevention. As favourable associations between greater adherence to the Portfolio Diet and other established cardiovascular risk factors beyond LDL-C, including other lipid, blood pressure and adiposity markers, were also seen, the benefit of the Portfolio Diet on lifetime cardiovascular health may be even more substantial than predicted in our model. Additionally, given that many young adults are highly conscious about climate change [70], their dietary choices may be influenced by environmental considerations [71, 72]. The Portfolio Diet, which includes mostly foods of low environmental impact [73], is a dietary pattern that can support both the planetary health and population health concerns faced by young adults today. Although some evidence exists to demonstrate the benefit of other dietary patterns, such as the DASH and Mediterranean diets, on cardiovascular risk factors in young adults [50–53], previous correlation analyses have demonstrated that these diets are not strongly correlated with the Portfolio Diet, despite sharing some similar foods [42]. This finding highlights the opportunity to provide individuals with a variety of dietary patterns for the management of cardiovascular risk factors, so they may choose a dietary pattern that best caters to their personal values and preferences. Importantly, while there were minimal subgroup differences by ethnicity, our assessment of adherence to the Portfolio Diet revealed that those with higher PDS were more likely to be Caucasian. Previous studies have characterised typical South Asian diets as high in saturated fat and both East and South Asian diets as high in refined carbohydrates [74–76], which may pose cultural barriers to adherence, as the Portfolio Diet is low in saturated fat and emphasizes the replacement of refined carbohydrates with whole grains as a source of viscous fibre. These potential barriers highlight the need for more culturally specific dietary recommendations to improve adherence across ethnically diverse populations.

## Conclusions

Higher PDS and intake of Portfolio Diet components was associated with lower LDL-C and several other established cardiovascular risk factors in an ethnoculturally diverse population of young adults. The observed associations align with current clinical practice guideline recommendations for the use of the Portfolio Diet for the prevention and management of CVD and suggest the potential for early adoption of a healthy dietary pattern to lower lifetime exposure to risk factors for the disease. Given the cross-sectional nature of this analysis, these results reflect associations rather than causal relationships. Therefore, future prospective studies are necessary to confirm these results and understand how early adoption of the Portfolio Diet in young adulthood may impact long-term cardiovascular health.

## Electronic supplementary material

Below is the link to the electronic supplementary material.

Supplementary Material 1

## Data Availability

The datasets analysed during the current study are available from the corresponding author upon reasonable request.
