# Supplementary material for: Portfolio diet and LDL-C in a young, multiethnic cohort: cross-sectional analyses with cumulative exposure modeling
Source: BMC Public Health. 2025 May 13;25:1761. doi: 10.1186/s12889-025-22479-9 (PMC12070585; doi:10.1186/s12889-025-22479-9)
Supplement: Supplementary file 1 — Supplementary Material 1 [file 12889_2025_22479_MOESM1_ESM.docx]

**Supplementary Table 1.** Scoring criteria for the Portfolio Diet Score from FFQ food items and mean daily intake^a^ for each quintile

| Component, servings/day | Food items in FFQ | Scoring criteria | | | | |
| --- | --- | --- | --- | --- | --- | --- |
|  |  | Q1 (1 point) | Q2 (2 points) | Q3 (3 points) | Q4 (4 points) | Q5 (5 points) |
| Plant protein | Tofu, soybeans or vegetable protein; peas or lima beans; lentils or beans, kidney, pinto, black-eyed, chickpeas, etc.; soymilk | 0.05 (0-0.10) | 0.17 (0.12-0.22) | 0.31 (0.24-0.44) | 0.60 (0.45-0.86) | 1.76 (0.88-11.50) |
| Viscous fibre | Cooked oatmeal; cooked oat bran; oat bran added to food; barley; fresh apples or pears; applesauce; oranges, tangerines, clementines; grapefruits strawberries, raspberries or blackberries; blueberries; okra; eggplant; Metamucil supplement | 0.21 (0-0.36) | 0.54 (0.36-0.71) | 0.95 (0.72-1.17) | 1.44 (1.18-1.83) | 3.05 (1.84-11.58) |
| Nuts | Peanut butter; almond butter; peanuts; almonds; walnuts; other nuts | 0.01 (0-0.04) | 0.10 (0.06-0.14) | 0.23 (0.16-0.34) | 0.53 (0.36-0.80) | 1.61 (0.81-5.32) |
| Phytosterols, mg/day | Estimated from all plant foods | 144 (54-179) | 208 (179-236) | 263 (236-294) | 331 (294-372) | 497 (372- 1,480) |
| MUFAs | Avocado; olive oil, added to food or bread; olive oil salad dressing | 0 (0-0) | 0.05 (0.02- 0.08) | 0.13 (0.10- 0.16) | 0.38 (0.18-0.51) | 1.41 (0.53-6.50) |
| Saturated fat and cholesterol^b^ | Whole milk; cream; ice cream; other cheese; cream cheese; eggs with yolk; bacon; poultry with skin; beef/pork hot dogs; chicken/turkey hot dogs; processed meats; lean hamburger; regular hamburger; mixed meals with red meat; pork; beef/lamb; beef liver; chicken liver; other organ meats; salami, bologna or other processed meat sandwiches; butter; pizza; cookies, home baked; cookies, other ready-made; brownies; doughnuts; pie, homemade; pie, ready made | 5.42 (4-12.22) | 3.37 (2.87-3.99) | 2.50 (2.16-2.87) | 1.82 (1.45-2.15) | 0.95 (0-1.44) |

Data are shown as mean (range).

FFQ, food frequency questionnaire; MUFAs, monounsaturated fatty acids; Q, quintiles.

^a^ All components reported as servings/day except for phytosterols (mg/day).

^b^ Higher quintile represent higher intake; however high intake and high quintile of saturated fat/cholesterol received lower scores.

**Supplementary Table 2**. Association between the Portfolio Diet Score and LDL-C estimated by the NIH, Martin and Vujovic equations

|  | n | T1 (low) | T2 (medium) | T3 (high) | P-trend^a^ | Per 1-PDS point | Per 8-PDS points | P-value^b^ |
| --- | --- | --- | --- | --- | --- | --- | --- | --- |
| **LDL-C (mmol/L)** | | | | | | | | |
| **NIH** | | | | | | | | |
| Unadjusted model | 1,490 | 2.34 (2.28, 2.39) | 2.29 (2.25, 2.32) | 2.24 (2.18, 2.29) | **0.023** | -0.010 (-0.017, -0.003) | -0.078 (-0.133, -0.023) | **0.006** |
| Multivariable model | 1,490 | 2.60 (2.36, 2.84) | 2.55 (2.32, 2.79) | 2.51 (2.27, 2.75) | **0.040** | -0.009 (-0.016, -0.002) | -0.073 (-0.130, -0.015) | **0.013** |
| **Martin** | | | | | | | | |
| Unadjusted model | 1,490 | 2.30 (2.25, 2.35) | 2.25 (2.22, 2.29) | 2.20 (2.15, 2.26) | **0.019** | -0.010 (-0.016, -0.003) | -0.077 (-0.131, -0.023) | **0.005** |
| Multivariable model | 1,490 | 2.54 (2.32, 2.76) | 2.50 (2.28, 2.72) | 2.45 (2.23, 2.68) | **0.038** | -0.009 (-0.016, -0.002) | -0.072 (-0.128, -0.016) | **0.012** |
| **Vujovic** | | | | | | | | |
| Unadjusted model | 1,490 | 2.43 (2.38, 2.48) | 2.38 (2.35, 2.42) | 2.33 (2.28, 2.39) | **0.023** | -0.010 (-0.016, -0.003) | -0.077 (-0.132, -0.022) | **0.006** |
| Multivariable model | 1,490 | 2.70 (2.46, 2.93) | 2.65 (2.42, 2.89) | 2.61 (2.37, 2.85) | **0.042** | -0.009 (-0.016, -0.002) | -0.071 (-0.128, -0.013) | **0.015** |

Associations between the PDS and LDL-C by tertiles and points of the PDS were assessed using multiple linear regressions. Data are marginal means (95% CIs) for each tertile and ß-coefficients (95% CIs) for continuous analyses by points. Prior to analysis, non-normal covariates (BMI and alcohol intake) were log-transformed or square-root transformed to an approximately normal distribution. Multivariable models were adjusted for sex (male, female), age (continuous), education (high school, some college/university, college/university degree, graduate degree), ethnicity (Caucasian, East Asian, South Asian and other), BMI (continuous), family history of CVD (yes/no), family history of diabetes (yes/no), hypertension status (yes/no), hypercholesterolemia status (yes/no), energy intake (continuous), smoking (current smoker [≥1/day], non-smoker), physical activity (continuous) and alcohol intake (continuous).

T1 (low), T2 (mid), and T3 (highest) tertiles of adherence to the Portfolio dietary pattern measured using the Portfolio Diet Score (PDS).

LDL-C, low-density lipoprotein cholesterol; PDS, Portfolio Diet Score; T, tertile.

To convert LDL-C mmol/L to mg/dL multiply by 38.67.

^a^ P for trend obtained from assigning the median value to each tertile.

^b^ P value obtained from continuous point increases in ß-coefficients.

**Supplementary Table 3.** LDL-C per 1-point higher PDS stratified by selected characteristics

|  | **N** | **Per 1-PDS point** |
| --- | --- | --- |
| **LDL-C (mmol/L)** | | |
| Sex |  |  |
| Male | 471 | -0.017 (-0.030, -0.004) |
| Female | 1,019 | -0.006 (-0.014, 0.003) |
| P-interaction |  | 0.093 |
|  |  |  |
| BMI |  |  |
| <25 kg/m^2^ | 1,171 | -0.009 (-0.016, -0.001) |
| ≥25 kg/m^2^ | 319 | -0.017 (-0.035, 0.001) |
| P-interaction |  | 0.225 |
|  |  |  |
| Ethnicity |  |  |
| Caucasian | 721 | -0.015 (-0.025, -0.005) |
| East Asian | 500 | -0.006 (-0.019, 0.007) |
| South Asian | 160 | 0.005 (-0.021, 0.030) |
| Other | 109 | -0.015 (-0.044, 0.014) |
| P-interaction |  | 0.632 |

Data are ß-coefficients (95% CIs). We used multiple linear regression to conduct this analysis. Multivariable models were adjusted for sex (male, female), age (continuous), education (high school, some college/university, college/university degree, graduate degree), ethnicity (Caucasian, East Asian, South Asian and other [individuals who reported belonging to ≥2 ethnocultural groups not included in the same category, Aboriginal Canadians, or Afro-Caribbeans]), BMI (continuous), family history of CVD (yes/no), family history of diabetes (yes/no), hypertension status (yes/no), hypercholesterolemia status (yes/no), energy intake (continuous), smoking (current smoker [≥1/day], non-smoker), physical activity (continuous) and alcohol intake (continuous). Prior to analysis, non-normal covariates (BMI and alcohol intake) were log-transformed or square-root transformed to an approximately normal distribution. BMI, body mass index; LDL-C, low-density lipoprotein cholesterol; PDS, Portfolio Diet Score.

**Supplementary Table 4.** Non-HDL-C per 1-point higher PDS stratified by selected characteristics

|  | **N** | **Per 1-PDS point** |
| --- | --- | --- |
| **Non-HDL-C (mmol/L)** | | |
| Sex |  |  |
| Male | 471 | -0.020 (-0.034, -0.005) |
| Female | 1,019 | -0.006 (-0.015, 0.003) |
| P-interaction |  | 0.111 |
|  |  |  |
| BMI |  |  |
| <25 kg/m^2^ | 1,171 | -0.008 (-0.017, 0.000) |
| ≥25 kg/m^2^ | 319 | -0.022 (-0.043, -0.001) |
| P-interaction |  | 0.144 |
|  |  |  |
| Ethnicity |  |  |
| Caucasian | 721 | -0.015 (-0.026, -0.004) |
| East Asian | 500 | -0.009 (-0.023, 0.005) |
| South Asian | 160 | 0.006 (-0.023, 0.035) |
| Other | 109 | -0.018 (-0.051, 0.014) |
| P-interaction |  | 0.394 |

Data are ß-coefficients (95% CIs). We used multiple linear regression to conduct this analysis. Multivariable models were adjusted for sex (male, female), age (continuous), education (high school, some college/university, college/university degree, graduate degree), ethnicity (Caucasian, East Asian, South Asian and other [individuals who reported belonging to ≥2 ethnocultural groups not included in the same category, Aboriginal Canadians, or Afro-Caribbeans]), BMI (continuous), family history of CVD (yes/no), family history of diabetes (yes/no), hypertension status (yes/no), hypercholesterolemia status (yes/no), energy intake (continuous), smoking (current smoker [≥1/day], non-smoker), physical activity (continuous) and alcohol intake (continuous). Prior to analysis, non-normal covariates (BMI and alcohol intake) were log-transformed or square-root transformed to an approximately normal distribution. BMI, body mass index; Non-HDL-C, non-high-density lipoprotein; PDS, Portfolio Diet Score.

**Supplementary Table 5.** Total cholesterol per 1-point higher PDS stratified by selected characteristics

|  | **N** | **Per 1-PDS point** |
| --- | --- | --- |
| **Total Cholesterol (mmol/L)** | | |
| Sex |  |  |
| Male | 471 | -0.023 (-0.038, -0.008) |
| Female | 1,020 | -0.006 (-0.016, 0.004) |
| P-interaction |  | **0.041** |
|  |  |  |
| BMI |  |  |
| <25 kg/m^2^ | 1,172 | -0.011 (-0.020, -0.001) |
| ≥25 kg/m^2^ | 319 | -0.017 (-0.038, 0.003) |
| P-interaction |  | 0.514 |
|  |  |  |
| Ethnicity |  |  |
| Caucasian | 721 | -0.013 (-0.025, -0.002) |
| East Asian | 501 | -0.013 (-0.028, 0.002) |
| South Asian | 160 | 0.003 (-0.026, 0.033) |
| Other | 109 | -0.019 (-0.054, 0.016) |
| P-interaction |  | 0.611 |

Data are ß-coefficients (95% CIs). We used multiple linear regression to conduct this analysis. Multivariable models were adjusted for sex (male, female), age (continuous), education (high school, some college/university, college/university degree, graduate degree), ethnicity (Caucasian, East Asian, South Asian and other [individuals who reported belonging to ≥2 ethnocultural groups not included in the same category, Aboriginal Canadians, or Afro-Caribbeans]), BMI (continuous), family history of CVD (yes/no), family history of diabetes (yes/no), hypertension status (yes/no), hypercholesterolemia status (yes/no), energy intake (continuous), smoking (current smoker [≥1/day], non-smoker), physical activity (continuous) and alcohol intake (continuous). Prior to analysis, non-normal covariates (BMI and alcohol intake) were log-transformed or square-root transformed to an approximately normal distribution. BMI, body mass index; PDS, Portfolio Diet Score.

**Supplementary Table 6.** HDL-C per 1-point higher PDS stratified by selected characteristics

|  | **N** | **Per 1-PDS point** |
| --- | --- | --- |
| **HDL-C (mmol/L)** | | |
| Sex |  |  |
| Male | 471 | -0.004 (-0.009, 0.002) |
| Female | 1,019 | 0.000 (-0.005, 0.005) |
| P-interaction |  | 0.222 |
|  |  |  |
| BMI |  |  |
| <25 kg/m^2^ | 1,171 | -0.002 (-0.007, 0.002) |
| ≥25 kg/m^2^ | 319 | 0.005 (-0.003, 0.012) |
| P-interaction |  | 0.114 |
|  |  |  |
| Ethnicity |  |  |
| Caucasian | 721 | 0.002 (-0.004, 0.007) |
| East Asian | 500 | -0.005 (-0.012, 0.002) |
| South Asian | 160 | -0.003 (-0.014, 0.008) |
| Other | 109 | -0.001(-0.016, 0.015) |
| P-interaction |  | 0.804 |

Data are ß-coefficients (95% CIs). We used multiple linear regression to conduct this analysis. Multivariable models were adjusted for sex (male, female), age (continuous), education (high school, some college/university, college/university degree, graduate degree), ethnicity (Caucasian, East Asian, South Asian and other [individuals who reported belonging to ≥2 ethnocultural groups not included in the same category, Aboriginal Canadians, or Afro-Caribbeans]), BMI (continuous), family history of CVD (yes/no), family history of diabetes (yes/no), hypertension status (yes/no), hypercholesterolemia status (yes/no), energy intake (continuous), smoking (current smoker [≥1/day], non-smoker), physical activity (continuous) and alcohol intake (continuous). Prior to analysis, non-normal covariates (BMI and alcohol intake) were log-transformed or square-root transformed to an approximately normal distribution. BMI, body mass index; HDL-C, high-density lipoprotein; PDS, Portfolio Diet Score.

**Supplementary Table 7.** Triglycerides per 1-point higher PDS stratified by selected characteristics

|  | **N** | **Per 1-PDS point** |
| --- | --- | --- |
| **Triglycerides (mmol/L)** | | |
| Sex |  |  |
| Male | 471 | -0.009 (-0.018, 0.000) |
| Female | 1,020 | -0.004 (-0.009, 0.002) |
| P-interaction |  | 0.746 |
|  |  |  |
| BMI |  |  |
| <25 kg/m^2^ | 1,172 | -0.019 (-0.007, 0.003) |
| ≥25 kg/m^2^ | 319 | -0.017 (-0.029, -0.004) |
| P-interaction |  | 0.062 |
|  |  |  |
| Ethnicity |  |  |
| Caucasian | 721 | -0.004 (-0.011, 0.002) |
| East Asian | 501 | -0.008 (-0.016, 0.001) |
| South Asian | 160 | 0.000 (-0.016, 0.016) |
| Other | 109 | -0.009 (-0.030, 0.013) |
| P-interaction |  | 0.347 |

Data are ß-coefficients (95% CIs). We used multiple linear regression to conduct this analysis. Multivariable models were adjusted for sex (male, female), age (continuous), education (high school, some college/university, college/university degree, graduate degree), ethnicity (Caucasian, East Asian, South Asian and other), BMI (continuous), family history of CVD (yes/no), family history of diabetes (yes/no), hypertension status (yes/no), hypercholesterolemia status (yes/no), energy intake (continuous), smoking (current smoker [≥1/day], non-smoker), physical activity (continuous) and alcohol intake (continuous). Prior to analysis, triglycerides and non-normal covariates (BMI and alcohol intake) were log-transformed or square-root transformed to an approximately normal distribution. Exponentiated ß-coefficients multiplied by the non-transformed mean were reported to facilitate interpretation. BMI, body mass index; PDS, Portfolio Diet Score.

**Supplementary Table 8.** CRP per 1-point higher PDS stratified by selected characteristics

|  | **N** | **Per 1-PDS point** |
| --- | --- | --- |
| **CRP (mg/L)** | | |
| Sex |  |  |
| Male | 470 | 0.000 (-0.025, 0.026) |
| Female | 1,024 | -0.016 (-0.034, 0.002) |
| P-interaction |  | 0.371 |
|  |  |  |
| BMI |  |  |
| <25 kg/m^2^ | 1,175 | -0.008 (-0.025, 0.008) |
| ≥25 kg/m^2^ | 319 | -0.021 (-0.058, 0.018) |
| P-interaction |  | 0.154 |
|  |  |  |
| Ethnicity |  |  |
| Caucasian | 723 | -0.003 (-0.025, 0.019) |
| East Asian | 502 | -0.027 (-0.051, -0.002) |
| South Asian | 160 | 0.007 (-0.041, 0.058) |
| Other | 109 | -0.016 (-0.073, 0.043) |
| P-interaction |  | 0.664 |

Data are ß-coefficients (95% CIs). We used multiple linear regression to conduct this analysis. Multivariable models were adjusted for sex (male, female), age (continuous), education (high school, some college/university, college/university degree, graduate degree), ethnicity (Caucasian, East Asian, South Asian and other), BMI (continuous), family history of CVD (yes/no), family history of diabetes (yes/no), hypertension status (yes/no), hypercholesterolemia status (yes/no), energy intake (continuous), smoking (current smoker [≥1/day], non-smoker), physical activity (continuous) and alcohol intake (continuous). Prior to analysis, CRP and non-normal covariates (BMI and alcohol intake) were log-transformed or square-root transformed to an approximately normal distribution. Exponentiated ß-coefficients multiplied by the non-transformed mean were reported to facilitate interpretation. BMI, body mass index; CRP, C-reactive protein; PDS, Portfolio Diet Score.

**Supplementary Table 9.** Fasting glucose per 1-point higher PDS stratified by selected characteristics

|  | **N** | **Per 1-PDS point** |
| --- | --- | --- |
| **Fasting glucose (mmol/L)** | | |
| Sex |  |  |
| Male | 471 | 0.000 (-0.007, 0.008) |
| Female | 1,025 | -0.002 (-0.006, 0.003) |
| P-interaction |  | 0.829 |
|  |  |  |
| BMI |  |  |
| <25 kg/m^2^ | 1,177 | -0.002 (-0.006, 0.002) |
| ≥25 kg/m^2^ | 319 | 0.001 (-0.009, 0.011 |
| P-interaction |  | 0.415 |
|  |  |  |
| Ethnicity |  |  |
| Caucasian | 724 | 0.003 (-0.002, 0.008) |
| East Asian | 503 | -0.008 (-0.015, -0.0002) |
| South Asian | 160 | -0.013 (-0.027, 0.0003) |
| Other | 109 | 0.015 (-0.002, 0.032) |
| P-interaction |  | **0.006** |

Data are ß-coefficients (95% CIs). We used multiple linear regression to conduct this analysis. Multivariable models were adjusted for sex (male, female), age (continuous), education (high school, some college/university, college/university degree, graduate degree), ethnicity (Caucasian, East Asian, South Asian and other), BMI (continuous), family history of CVD (yes/no), family history of diabetes (yes/no), hypertension status (yes/no), hypercholesterolemia status (yes/no), energy intake (continuous), smoking (current smoker [≥1/day], non-smoker), physical activity (continuous) and alcohol intake (continuous). Prior to analysis, non-normal covariates (BMI and alcohol intake) were log-transformed or square-root transformed to an approximately normal distribution. BMI, body mass index; PDS, Portfolio Diet Score.

**Supplementary Table 10.** SBP per 1-point higher PDS stratified by selected characteristics

|  | **N** | **Per 1-PDS point** |
| --- | --- | --- |
| **SBP (mmHg)** | | |
| Sex |  |  |
| Male | 475 | -0.080 (-0.266, 0.106) |
| Female | 1,032 | -0.196 (-0.315, -0.077) |
| P-interaction |  | 0.092 |
|  |  |  |
| BMI |  |  |
| <25 kg/m^2^ | 1,185 | -0.171 (-0.283, -0.059) |
| ≥25 kg/m^2^ | 322 | -0.167 (-0.408, 0.074) |
| P-interaction |  | 0.832 |
|  |  |  |
| Ethnicity |  |  |
| Caucasian | 731 | -0.226 (-0.367, -0.085) |
| East Asian | 507 | -0.138 (-0.325, 0.049) |
| South Asian | 160 | -0.046 (-0.357, 0.265) |
| Other | 109 | -0.097 (-0.485, 0.290) |
| P-interaction |  | 0.487 |

Data are ß-coefficients (95% CIs). We used multiple linear regression to conduct this analysis. Multivariable models were adjusted for sex (male, female), age (continuous), education (high school, some college/university, college/university degree, graduate degree), ethnicity (Caucasian, East Asian, South Asian and other), BMI (continuous), family history of CVD (yes/no), family history of diabetes (yes/no), hypertension status (yes/no), hypercholesterolemia status (yes/no), energy intake (continuous), smoking (current smoker [≥1/day], non-smoker), physical activity (continuous) and alcohol intake (continuous). Prior to analysis, non-normal covariates (BMI and alcohol intake) were log-transformed or square-root transformed to an approximately normal distribution. BMI, body mass index; SBP, systolic blood pressure; PDS, Portfolio Diet Score.

**Supplementary Table 11.** DBP per 1-point higher PDS stratified by selected characteristics

|  | **N** | **Per 1-PDS point** |
| --- | --- | --- |
| **DBP (mmHg)** | | |
| Sex |  |  |
| Male | 475 | -0.109 (-0.267, 0.049) |
| Female | 1,032 | -0.157 (-0.261, -0.052) |
| P-interaction |  | 0.884 |
|  |  |  |
| BMI |  |  |
| <25 kg/m^2^ | 1,185 | -0.115 (-0.211, -0.019) |
| ≥25 kg/m^2^ | 322 | -0.261 (-0.477, -0.044) |
| P-interaction |  | 0.214 |
|  |  |  |
| Ethnicity |  |  |
| Caucasian | 731 | -0.180 (-0.300, -0.059) |
| East Asian | 507 | -0.125 (-0.293, 0.043) |
| South Asian | 160 | 0.065 (-0.198, 0.329) |
| Other | 109 | -0.257 (-0.591, 0.077) |
| P-interaction |  | 0.195 |

Data are ß-coefficients (95% CIs). We used multiple linear regression to conduct this analysis. Multivariable models were adjusted for sex (male, female), age (continuous), education (high school, some college/university, college/university degree, graduate degree), ethnicity (Caucasian, East Asian, South Asian and other), BMI (continuous), family history of CVD (yes/no), family history of diabetes (yes/no), hypertension status (yes/no), hypercholesterolemia status (yes/no), energy intake (continuous), smoking (current smoker [≥1/day], non-smoker), physical activity (continuous) and alcohol intake (continuous). Prior to analysis, non-normal covariates (BMI and alcohol intake) were log-transformed or square-root transformed to an approximately normal distribution. BMI; body mass index; DBP, diastolic blood pressure; PDS, Portfolio Diet Score.

**Supplementary Table 12.** BMI per 1-point higher PDS stratified by selected characteristics

|  | **N** | **Per 1-PDS point** |
| --- | --- | --- |
| **BMI (kg/m^2^)** | | |
| Sex |  |  |
| Male | 475 | -0.028 (-0.092, 0.037) |
| Female | 1,032 | -0.044 (-0.083, -0.006) |
| P-interaction |  | 0.875 |
|  |  |  |
| Ethnicity |  |  |
| Caucasian | 731 | -0.089 (-0.134, -0.044) |
| East Asian | 507 | 0.032 (-0.020, 0.084) |
| South Asian | 160 | 0.033 (-0.089, 0.155) |
| Other | 109 | -0.009 (-0.195, 0.178) |
| P-interaction |  | 0.066 |

Data are ß-coefficients (95% CIs). We used multiple linear regression to conduct this analysis. Multivariable models were adjusted for sex (male, female), age (continuous), education (high school, some college/university, college/university degree, graduate degree), ethnicity (Caucasian, East Asian, South Asian and other), family history of CVD (yes/no), family history of diabetes (yes/no), hypertension status (yes/no), hypercholesterolemia status (yes/no), energy intake (continuous), smoking (current smoker [≥1/day], non-smoker), physical activity (continuous) and alcohol intake (continuous). Prior to analysis, BMI and non-normal covariates (alcohol intake) were log-transformed or square-root transformed to an approximately normal distribution. Exponentiated ß-coefficients multiplied by the non-transformed mean were reported to facilitate interpretation. BMI, body mass index; PDS, Portfolio Diet Score.

**Supplementary Table 13.** Waist circumference per 1-point higher PDS stratified by selected characteristics

|  | **N** | **Per 1-PDS point** |
| --- | --- | --- |
| **Waist Circumference (cm)** | | |
| Sex |  |  |
| Male | 475 | -0.130 (-0.289, 0.028) |
| Female | 1,032 | -0.084 (-0.174, 0.006) |
| P-interaction |  | 0.588 |
|  |  |  |
| Ethnicity |  |  |
| Caucasian | 731 | -0.214 (-0.319, -0.110) |
| East Asian | 507 | 0.036 (-0.091, 0.164) |
| South Asian | 160 | 0.028 (-0.262, 0.320) |
| Other | 109 | 0.074 (-0.368, 0.518) |
| P-interaction |  | 0.103 |

Data are ß-coefficients (95% CIs). We used multiple linear regression to conduct this analysis. Multivariable models were adjusted for sex (male, female), age (continuous), education (high school, some college/university, college/university degree, graduate degree), ethnicity (Caucasian, East Asian, South Asian and other), family history of CVD (yes/no), family history of diabetes (yes/no), hypertension status (yes/no), hypercholesterolemia status (yes/no), energy intake (continuous), smoking (current smoker [≥1/day], non-smoker), physical activity (continuous) and alcohol intake (continuous). Prior to analysis, waist circumference and non-normal covariates (alcohol intake) were log-transformed or square-root transformed to an approximately normal distribution. Exponentiated ß-coefficients multiplied by the non-transformed mean were reported to facilitate interpretation. PDS, Portfolio Diet Score.

**Supplementary Table 14.** Body Weight per 1-point higher PDS stratified by selected characteristics

|  | **N** | **Per 1-PDS point** |
| --- | --- | --- |
| **Body Weight (kg)** | | |
| Sex |  |  |
| Male | 475 | -0.176 (-0.380, 0.029) |
| Female | 1,032 | -0.113 (-0.235, 0.009) |
| P-interaction |  | 0.288 |
|  |  |  |
| Ethnicity |  |  |
| Caucasian | 731 | -0.251 (-0.395, -0.106) |
| East Asian | 507 | 0.077 (-0.093, 0.247) |
| South Asian | 160 | -0.037 (-0.412, 0.340) |
| Other | 109 | -0.055 (-0.594, 0.487) |
| P-interaction |  | 0.091 |

Data are ß-coefficients (95% CIs). We used multiple linear regression to conduct this analysis. Multivariable models were adjusted for sex (male, female), age (continuous), education (high school, some college/university, college/university degree, graduate degree), ethnicity (Caucasian, East Asian, South Asian and other), family history of CVD (yes/no), family history of diabetes (yes/no), hypertension status (yes/no), hypercholesterolemia status (yes/no), energy intake (continuous), smoking (current smoker [≥1/day], non-smoker), physical activity (continuous) and alcohol intake (continuous). Prior to analysis, body weight and non-normal covariates (alcohol intake) were log-transformed or square-root transformed to an approximately normal distribution. Exponentiated ß-coefficients multiplied by the non-transformed mean were reported to facilitate interpretation. PDS, Portfolio Diet Score.

**Supplementary Table 15.** FMI per 1-point higher PDS stratified by selected characteristics

|  | **N** | **Per 1-PDS point** |
| --- | --- | --- |
| **FMI (kg/m^2^)** | | |
| Sex |  |  |
| Male | 475 | -0.017 (-0.058, 0.024) |
| Female | 1,032 | -0.022 (-0.041, -0.003) |
| P-interaction |  | 0.809 |
|  |  |  |
| Ethnicity |  |  |
| Caucasian | 731 | -0.048 (-0.072, -0.024) |
| East Asian | 507 | 0.016 (-0.013, 0.045) |
| South Asian | 160 | 0.023 (-0.045, 0.093) |
| Other | 109 | 0.011 (-0.087, 0.112) |
| P-interaction |  | 0.057 |

Data are ß-coefficients (95% CIs). We used multiple linear regression to conduct this analysis. Multivariable models were adjusted for sex (male, female), age (continuous), education (high school, some college/university, college/university degree, graduate degree), ethnicity (Caucasian, East Asian, South Asian and other), family history of CVD (yes/no), family history of diabetes (yes/no), hypertension status (yes/no), hypercholesterolemia status (yes/no), energy intake (continuous), smoking (current smoker [≥1/day], non-smoker), physical activity (continuous) and alcohol intake (continuous). Prior to analysis, FMI and non-normal covariates (alcohol intake) were log-transformed or square-root transformed to an approximately normal distribution. Exponentiated ß-coefficients multiplied by the non-transformed mean were reported to facilitate interpretation. FMI, fat mass index; PDS, Portfolio Diet Score.

**Supplementary Table 16.** Cardiovascular risk factors according to servings of the Portfolio Diet components

|  | Plant Protein | Viscous Fibre | Nuts | MUFA | Phytosterols | Saturated Fat and Cholesterol |
| --- | --- | --- | --- | --- | --- | --- |
|  | Per serving | Per serving | Per serving | Per serving | Per 25g^a^ | Per serving |
| **Lipids** | | | | | | |
| **LDL-C (mmol/L)** | | | | | | |
| Unadjusted model | -0.047 (-0.087, -0.007) | -0.015 (-0.043, 0.014) | -0.059 (-0.105, -0.013) | -0.012 (-0.059, 0.036) | -0.008 (-0.014, -0.002) | 0.016 (-0.004, 0.036) |
| P-value | **0.020** | 0.311 | **0.012** | 0.623 | **0.013** | 0.115 |
| Multivariable model | -0.051 (-0.090, -0.011) | -0.015 (-0.044, 0.014) | -0.049 (-0.096, -0.003) | -0.012 (-0.060, 0.036) | -0.013 (-0.021, -0.005) | 0.028 (0.004, 0.051) |
| P-value | **0.012** | 0.318 | **0.038** | 0.627 | **0.002** | **0.020** |
| **Non-HDL-C (mmol/L)** | | | | | | |
| Unadjusted model | -0.052 (-0.096, -0.008) | -0.021 (-0.052, 0.011) | -0.074 (-0.126, -0.023) | -0.019 (-0.072, 0.034) | -0.008 (-0.015, -0.002) | 0.016 (-0.007, 0.038) |
| P-value | **0.022** | 0.198 | **0.005** | 0.477 | **0.016** | 0.170 |
| Multivariable model | -0.054 (-0.097, -0.010) | -0.021 (-0.053, 0.011) | -0.062 (-0.113, -0.010) | -0.023 (-0.075, 0.030) | -0.015 (-0.024, -0.006) | 0.023 (-0.003, 0.049) |
| P-value | **0.016** | 0.192 | **0.019** | 0.399 | **0.001** | 0.081 |
| **Total cholesterol (mmol/L)** | | | | | | |
| Unadjusted model | -0.052 (-0.098, -0.006) | -0.011 (-0.044, 0.022) | -0.062 (-0.116, -0.008) | -0.014 (-0.070, 0.041) | -0.013 (-0.020, -0.006) | 0.010 (-0.013, 0.034) |
| P-value | **0.027** | 0.530 | **0.025** | 0.619 | **0.001** | 0.390 |
| Multivariable model | -0.057 (-0.103, -0.011) | -0.018 (-0.051, 0.016) | -0.057 (-0.111, -0.002) | -0.013 (-0.069, 0.042) | -0.017 (-0.026, -0.007) | 0.036 (0.009, 0.063) |
| P-value | **0.016** | 0.307 | **0.041** | 0.637 | **0.001** | **0.010** |
| **HDL-C (mmol/L)** | | | | | | |
| Unadjusted model | 0.000 (-0.023, 0.024) | 0.009 (-0.008, 0.026) | 0.013 (-0.014, 0.040) | 0.006 (-0.022, 0.034) | -0.004 (-0.008, -0.001) | -0.005 (-0.017, 0.007) |
| P-value | 0.988 | 0.292 | 0.348 | 0.692 | **0.021** | 0.417 |
| Multivariable model | -0.003 (-0.024, 0.018) | 0.002 (-0.013, 0.017) | 0.005 (-0.020, 0.029) | 0.009 (-0.016, 0.034) | -0.002 (-0.006, 0.002) | 0.013 (0.001, 0.025) |
| P-value | 0.798 | 0.778 | 0.700 | 0.468 | 0.365 | **0.039** |
| **Triglycerides (mmol/L)^b^** | | | | | | |
| Unadjusted model | -0.030 (-0.054, -0.005) | -0.018 (-0.036, 0.000) | -0.046 (-0.074, -0.017) | -0.026 (-0.055, 0.005) | -0.004 (-0.008, 0.000) | 0.002 (-0.011, 0.016) |
| P-value | **0.021** | 0.055 | **0.002** | 0.097 | **0.035** | 0.721 |
| Multivariable model | -0.025 (-0.050, 0.000) | -0.018 (-0.036, 0.001) | -0.040 (-0.069, -0.010) | -0.033 (-0.063, -0.002) | -0.006 (-0.012, -0.001) | -0.001 (-0.016, 0.014) |
| P-value | 0.053 | 0.062 | **0.009** | **0.036** | **0.020** | 0.884 |
| **Inflammation** | | | | | | |
| **CRP (mg/L)^b^** | | | | | | |
| Unadjusted model | -0.084 (-0.163, 0.000) | -0.016 (-0.076, 0.047) | -0.073 (-0.165, 0.026) | -0.010 (-0.110, 0.098) | -0.015 (-0.028, -0.002) | -0.010 (-0.054, 0.035) |
| P-value | 0.050 | 0.605 | 0.146 | 0.850 | **0.027** | 0.649 |
| Multivariable model | -0.022 (-0.100, 0.061) | -0.034 (-0.091, 0.025) | -0.086 (-0.172, 0.007) | -0.096 (-0.183, -0.001) | -0.007 (-0.024, 0.010) | 0.006 (-0.042, 0.055) |
| P-value | 0.591 | 0.256 | 0.070 | **0.048** | 0.393 | 0.815 |
| **Glycemic control** | | | | | | |
| **Fasting glucose (mmol/L)** | | | | | | |
| Unadjusted model | -0.010 (-0.032, 0.012) | -0.017 (-0.033, -0.001) | -0.009 (-0.035, 0.016) | 0.001 (-0.026, 0.027) | -0.001 (-0.004, 0.003) | 0.006 (-0.005, 0.017) |
| P-value | 0.371 | **0.034** | 0.481 | 0.964 | 0.691 | 0.318 |
| Multivariable model | -0.007 (-0.028, 0.015) | -0.009 (-0.024, 0.007) | 0.006 (-0.020, 0.031) | 0.003 (-0.023, 0.029) | -0.002 (-0.006, 0.003) | 0.002 (-0.011, 0.014) |
| P-value | 0.541 | 0.263 | 0.667 | 0.822 | 0.501 | 0.807 |
| **Blood pressure** | | | | | | |
| **SBP (mmHg)** | | | | | | |
| Unadjusted model | -1.047 (-1.747, -0.347) | -0.528 (-1.028, -0.029) | 0.443 (-0.372, 1.257) | 0.481 (-0.350, 1.311) | 0.071 (-0.036, 0.179) | 1.113 (0.762, 1.465) |
| P-value | **0.003** | **0.038** | 0.287 | 0.257 | 0.193 | **<0.001** |
| Multivariable model | -0.854 (-1.403, -0.305) | -0.342 (-0.743, 0.059) | 0.094 (-0.554, 0.742) | -0.271 (-0.931, 0.388) | -0.110 (-0.223, 0.004) | 0.451 (0.128, 0.774) |
| P-value | **0.002** | 0.095 | 0.777 | 0.420 | 0.059 | **0.006** |
| **DBP (mmHg)** | | | | | | |
| Unadjusted model | -0.492 (-0.979, -0.005) | -0.374 (-0.720, -0.027) | -0.173 (-0.739, 0.392) | 0.216 (-0.361, 0.792) | -0.018 (-0.093, 0.057) | 0.356 (0.109, 0.602) |
| P-value | **0.048** | **0.035** | 0.548 | 0.463 | 0.632 | **0.005** |
| Multivariable model | -0.355 (-0.833, 0.123) | -0.375 (-0.723, -0.027) | -0.138 (-0.700, 0.424) | -0.015 (-0.587, 0.558) | -0.039 (-0.138, 0.060) | 0.345 (0.064, 0.625) |
| P-value | 0.146 | **0.035** | 0.630 | 0.960 | 0.442 | **0.016** |
| **Markers of adiposity** | | | | | | |
| **BMI (kg/m^2^)^b^** | | | | | | |
| Unadjusted model | -0.231 (-0.430, -0.031) | 0.033 (-0.110, 0.178) | -0.255 (-0.485, -0.022) | 0.283 (0.043, 0.526) | -0.018 (-0.049, 0.013) | 0.062 (-0.040, 0.165) |
| P-value | **0.024** | 0.651 | **0.032** | **0.021** | 0.254 | 0.233 |
| Multivariable model | -0.130 (-0.311, 0.052) | 0.025 (-0.108, 0.159) | -0.359 (-0.568, -0.147) | 0.072 (-0.146, 0.293) | -0.048 (-0.085, -0.010) | -0.009 (-0.116, 0.099) |
| P-value | 0.162 | 0.714 | **0.001** | 0.518 | **0.013** | 0.872 |
| **Waist circumference (cm)^b^** | | | | | | |
| Unadjusted model | -0.462 (-0.984, 0.064) | -0.119 (-0.493, 0.258) | -0.386 (-0.993, 0.226) | 0.807 (0.180, 1.440) | 0.020 (-0.061, 0.101) | 0.444 (0.176, 0.712) |
| P-value | 0.085 | 0.536 | 0.216 | **0.012** | 0.628 | **0.001** |
| Multivariable model | -0.217 (-0.649, 0.217) | 0.006 (-0.310, 0.324) | -0.827 (-1.327, -0.324) | 0.191 (-0.328, 0.714) | -0.108 (-0.198, -0.019) | -0.055 (-0.310, 0.201) |
| P-value | 0.325 | 0.969 | **0.001** | 0.472 | **0.017** | 0.673 |
| **Body weight (kg)^b^** | | | | | | |
| Unadjusted model | -0.867 (-1.607, -0.118) | 0.052 (-0.485, 0.593) | 0.104 (-0.769, 0.988) | 1.401 (0.496, 2.319) | 0.058 (-0.058, 0.174) | 0.924 (0.539, 1.311) |
| P-value | **0.023** | 0.851 | 0.817 | **0.002** | 0.328 | **<0.001** |
| Multivariable model | -0.496 (-1.067, 0.082) | 0.121 (-0.300, 0.546) | -0.754 (-1.421, -0.080) | 0.155 (-0.537, 0.855) | -0.171 (-0.290, -0.052) | 0.104 (-0.236, 0.447) |
| P-value | 0.092 | 0.573 | **0.029** | 0.661 | **0.005** | 0.548 |
| **FMI (kg/m^2^)^b^** | | | | | | |
| Unadjusted model | -0.093 (-0.212, 0.030) | 0.124 (0.035, 0.214) | -0.136 (-0.274, 0.005) | 0.173 (0.026, 0.325) | -0.026 (-0.045, -0.007) | -0.076 (-0.137, -0.014) |
| P-value | 0.137 | **0.006** | 0.058 | **0.021** | **0.006** | **0.016** |
| Multivariable model | -0.061 (-0.159, 0.039) | 0.019 (-0.054, 0.092) | -0.199 (-0.311, -0.085) | 0.050 (-0.070, 0.173) | -0.025 (-0.046, -0.004) | -0.006 (-0.065, 0.053) |
| P-value | 0.229 | 0.617 | **0.001** | 0.414 | **0.018** | 0.844 |

Associations between the Portfolio Diet components and cardiovascular risk factors by servings of each component were assessed using multiple linear regressions. A serving of phytosterols was estimated to be 25mg from the mean phytosterol content of plant foods included in the FFQ. Data are ß-coefficients (95% CIs). Prior to analysis, non-normal outcomes and covariates (triglycerides, CRP, BMI, waist circumference, body weight, FMI and alcohol intake) were log-transformed or square-root transformed to an approximately normal distribution. Multivariable models were adjusted for sex (male, female), age (continuous), education (high school, some college/university, college/university degree, graduate degree), ethnicity (Caucasian, East Asian, South Asian and other [individuals who reported belonging to ≥2 ethnocultural groups not included in the same category, Aboriginal Canadians, or Afro-Caribbeans]), BMI (continuous; not included in models for markers of adiposity), family history of CVD (yes/no), family history of diabetes (yes/no), hypertension status (yes/no), hypercholesterolemia status (yes/no), energy intake (continuous), smoking (current smoker [≥1/day], non-smoker), physical activity (continuous) and alcohol intake (continuous).

BMI, body mass index; DBP, diastolic blood pressure; FMI, fat mass index; HDL-C, high-density lipoprotein cholesterol; LDL-C, low-density lipoprotein cholesterol; PDS, Portfolio Diet Score; SBP, systolic blood pressure; T, tertile; WC, waist circumference.

To convert LDL-C, non-HDL-C, total cholesterol or HDL-C from mmol/L to mg/dL multiply by 38.67.

To convert triglycerides from mmol/L to mg/dL multiply by 88.57.

To convert CRP from mg/L to mg/dL divide by 10.

To convert fasting glucose from mmol/L to mg/dL multiple by 18.018.

^a^ 1-serving of phytosterols was estimated to be 25mg from the mean phytosterol content of plant foods included in the FFQ

^b^ Exponentiated ß coefficients multiplied by the non-transformed mean were reported to facilitate interpretation of log-transformed variables. P-values provided are from the log-transformed analysis.
